# Profile of language abilities in a sample of adults with developmental disorders

**DOI:** 10.1002/dys.1672

**Published:** 2020-11-17

**Authors:** Abigail R. Bradshaw, Zoe V. J. Woodhead, Paul A. Thompson, Dorothy V. M. Bishop

**Affiliations:** ^1^ Department of Experimental Psychology University of Oxford Oxford UK

**Keywords:** adults, developmental coordination disorder, developmental disorders, dyslexia, language

## Abstract

This study investigated the profile of language abilities in a sample of high‐achieving English speaking adults with developmental disorders. Ninety‐seven adult participants were recruited: 49 with a dyslexia diagnosis (dyslexic group), 16 with a diagnosis of a different developmental disorder including dyspraxia, autism and SpLD (non‐dyslexic developmental disorder group) and 32 with no diagnosis (non‐disordered group). Dyslexic and non‐dyslexic developmental disorder groups demonstrated similar impairments across measures of word reading, working memory, processing speed and oral language. Dyslexic participants showed the usual pattern of impaired phonological skills but spared non‐verbal intelligence and vocabulary. There were also some suggestions of impaired structural oral language skills in this group. A data‐driven clustering analysis found that diagnosis was not a reliable predictor of similarity between cases, with diagnostic categories split between data‐driven clusters. Overall, the findings indicate that high‐achieving adults with developmental disorders do demonstrate impairments that are likely to affect success in higher education, but that support needs should be assessed on a case‐by‐case basis, rather than according to diagnostic label.

## 1

**Practitioner points**

- High achieving adults with developmental disorders still demonstrate a range of impairments in memory, language and processing skills relative to their peer group.
- Impairments on measures of structural language skills are found in both adults with dyslexia and other related developmental disorders.
- A clustering analysis indicated that diagnostic labels may not always be informative for predicting type and level of impairments in adults.
- Support needs for adults with developmental disorders in higher education should be assessed on a case‐by‐case basis.

The term “developmental disorder” refers to disorders that emerge early on in life that are assumed to involve abnormal neurodevelopment, with complex or unknown multifactorial origins. Disorders that come under this term include developmental dyslexia, developmental language disorder (DLD, also known as specific language impairment), dyspraxia (also known as developmental coordination disorder or DCD), autism and attention deficit hyperactivity disorder (ADHD).

Although such disorders are first recognized early in development and can in many cases improve with age, they often have persistent effects into adulthood. Large amounts of research have gone into characterizing the profiles of impairment in children with developmental disorders, but few studies have followed cases through to adulthood. This limits our understanding of how developmental disorders may continue to have detrimental effects in adults, even in those who appear to be “high achieving.” Furthermore, it is unclear to what extent different diagnostic labels, applied in the context of measures taken in childhood, have lasting validity in adulthood.

The current study aimed to provide some insights into the nature of developmental disorders in adulthood, with a particular focus on language abilities. Two key questions were considered: (1) Do high achieving adults with diagnoses of developmental disorders demonstrate persisting deficits on measures of language and memory? and (2) Do diagnostic categories in adulthood correspond to discrete patterns of impairment?

### 1.1 Language abilities in adults with developmental disorders

Understanding the persisting areas of difficulty shown by adults with developmental disorders is relevant for their support needs in higher education. More and more adults with developmental disorders are entering higher education; in particular, there have been large increases in the number of dyslexic students entering University (Henderson, 2017; Mortimore & Crozier, 2006). This raises the question as to the level of support such individuals should receive during their higher education. That is, do adults who received a diagnosis of a developmental disorder as a child still experience difficulty within their affected areas? It has been reported that compensation to achieve reading skills in the normal range by adulthood occurs in around 25% of individuals who experience reading difficulties as a child (Lefly & Pennington, 1991). Conversely, a recent study by Bergey, Parrila, and Deacon (2018) reported that students entering a Canadian university with a history of reading difficulties demonstrated lower academic self‐efficacy and achieved lower grades than their typically reading peers. This suggests that these high achieving individuals may still be at a disadvantage when entering higher education.

One area of weakness that is commonly reported across different developmental disorders in childhood is difficulty with language skills. Many studies have found that significant proportions of children with a range of developmental disorders would also meet criteria for language impairment, and vice versa. For example, in one study, just over half of a sample of children with dyslexia scored more than one standard deviation below the mean of the CELF‐R (Clinical Evaluation of Language Fundamentals‐Revised; Semel, Wiig, & Secord, 1987), an instrument that is frequently used to diagnose DLD (McArthur, Hogben, Edwards, Heath, & Mengler, 2000). In a sample of children with DLD, about a third was found to also meet criteria for dyspraxia (Flapper & Schoemaker, 2013). Language difficulties are also common in autism; in particular, deficits in structural language similar to those seen in DLD are often reported (Tager‐Flusberg & Joseph, 2003).

This raises the question of whether similar language difficulties are also apparent in adults with these developmental disorders. A seminal meta‐analysis by Swanson and Hsieh (2009) found that adults with reading disabilities demonstrated a range of deficits on standardized measures that extended beyond a phonological core deficit, for example with additional impairments on naming speed and verbal memory. A recent study by Wiseheart and Altmann (2018) reported that high achieving dyslexic university students were impaired on a spoken sentence generation task relative to controls, being slower to respond and constructing sentences that were less precise and less fluent. Other studies have reported a sparing of vocabulary skills (e.g., Hatcher, Snowling, & Griffiths, 2002), with one study even reporting superior performance on a measure of vocabulary depth in French speaking dyslexic students compared to controls (Cavalli et al., 2016). A strong vocabulary may offer a compensatory strategy for reading that can enable dyslexics to access and cope with the large amounts of written information they will encounter during a university degree.

These studies suggest that a number of language difficulties not limited to the traditional core phonological deficit can persist in adults with dyslexia, even in those who have successfully reached higher education. This suggests the need for further study of how a wide range of language skills may be affected in adults with dyslexia, as well as in adults with other developmental disorders that are known to show language difficulties in childhood.

### 1.2 The validity of diagnostic categories

Research on developmental disorders typically focuses on single diagnostic groups, with strict exclusionary criteria for recruitment of “pure” cases of a specific disorder. However, in childhood, comorbidity across developmental disorders appears to be the rule rather than the exception (Kaplan, Dewey, Crawford, & Wilson, 2001; McGrath et al., 2008; Watemberg, Waiserberg, Zuk, & Lerman‐Sagie, 2007). In a study of 179 school‐aged children using criteria for seven disorders, Kaplan et al. (2001) found that 50% met criteria for at least two developmental disorders. To take dyspraxia as an example, studies have found that up to 50% of individuals in samples meet criteria for ADHD (Kadesjö & Gillberg, 1999), and more than half show reading disability (Kaplan, Wilson, Dewey, & Crawford, 1998).

This raises the question of how we should interpret such comorbidity. That is, if disorders demonstrate overlapping patterns of behaviour, are the distinctions made between them valid? For example, overlap between DLD and developmental dyslexia has been used to argue that these may be best viewed as different manifestations of a common underlying pathology, rather than as distinct disorders (Kamhi & Catts, 1986). Others have taken this even further, to argue that the concept of different developmental disorders as discrete entities should be abandoned entirely. Using discriminant function and clustering analyses on data from children with a wide variety of developmental disorders, Dyck, Piek, and Patrick (2011) found no evidence for clear discontinuity between different diagnostic categories, or indeed between the disorders and typically developing cases. They argued that these disorders should be viewed as locations in a continuously varying multidimensional space, rather than as discrete entities (Kendell & Jablensky, 2003).

**FIGURE 1:**
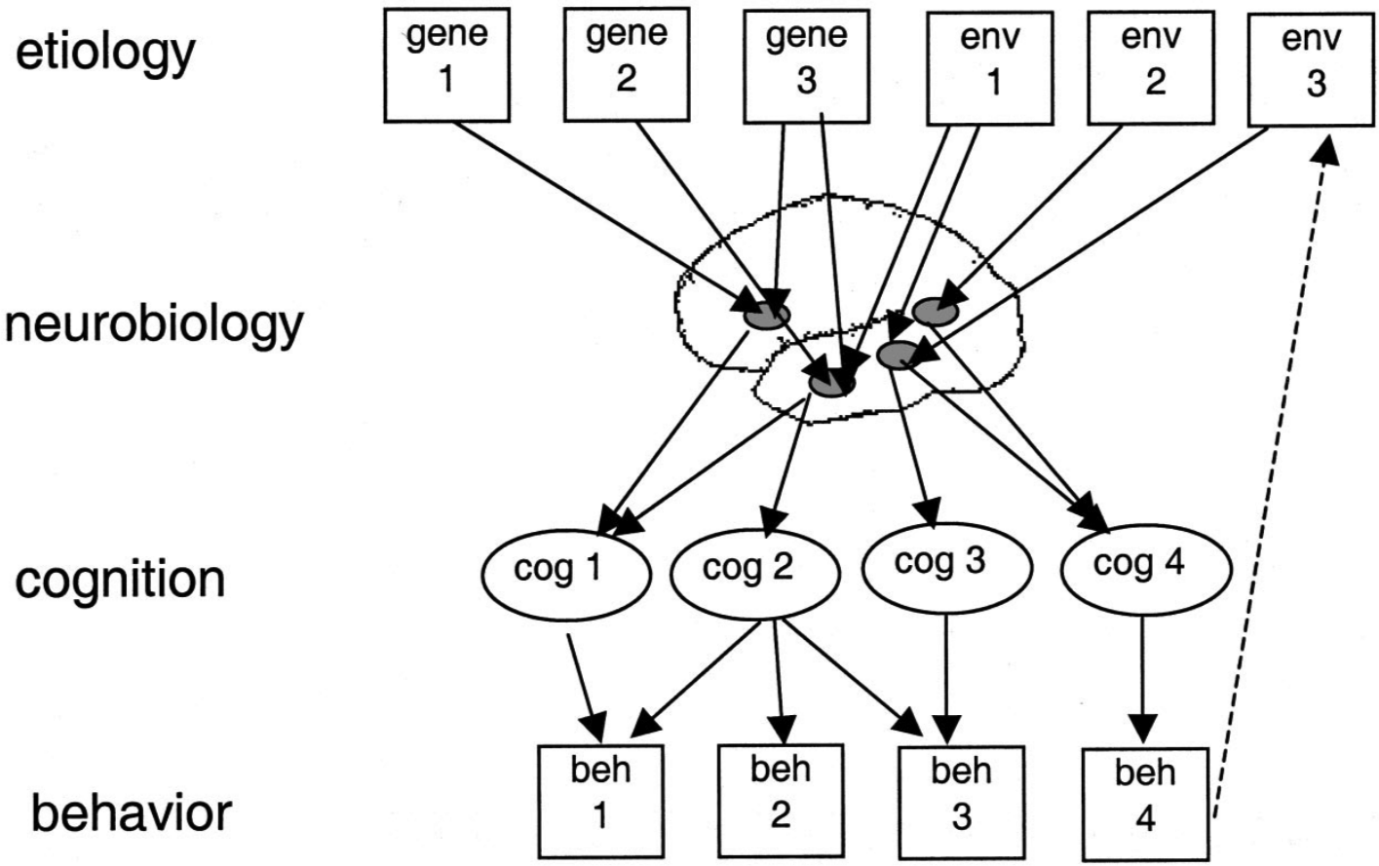
Multi‐level framework of causation for developmental disorders. Diagram illustrates links between different levels of causality, and how a causal factor at one level (e.g., cognition) may be associated with multiple outcomes at another level (e.g., behaviour). beh, behaviour; cog, cognition; Env, environment. Reprinted with permission from Bishop and Snowling (2004)

However, it is important to caution against the assumption that shared patterns of behaviour necessitate shared causal origins. To illustrate this, we can draw on a causal framework that considers disorders at multiple levels (see Figure 1). This makes distinctions between the levels of observed behaviour, cognitive processes, neurobiology and aetiology, that is, genes and environment (Bishop & Rutter, 2009; Bishop & Snowling, 2004). Within this framework, the relationships between levels are not one‐to‐one, such that a shared origin at one level need not result in shared patterns at another; conversely, different origins at higher levels (e.g., different genetic influences) could result in shared patterns at lower levels (e.g., shared cognitive impairments). In this way, it is important to exercise caution when interpreting overlap between impairments across developmental disorders.

More recently, a small number of studies have applied unsupervised machine learning approaches to data obtained from heterogeneous samples containing a range of difficulties and diagnostic labels (Archibald, Cardy, Ansari, Olino, & Joanisse, 2019; Archibald, Cardy, Joanisse, & Ansari, 2013; Astle, Bathelt, & Holmes, 2019; Bathelt et al., 2018; see Hong, 2018 for an editorial comment). The key feature of this approach is that the algorithm is not fed pre‐specified group memberships, but instead attempts to derive sub‐groups based on similarity structure within the data. This research has highlighted that such data‐driven “clusters” do not necessarily correspond well to diagnostic labels of cases, but nevertheless cluster‐membership can reliably predict behavioural outcomes and patterns of structural brain connectivity (Astle et al., 2019).

To our knowledge, such data‐driven clustering analyses have not yet been applied to data from adults with developmental disorders. A similar failure to find that diagnostic labels predict data‐driven cluster membership in adults would have important implications for practise, suggesting that basing decisions for support provided by university disability services on diagnostic labels alone may not be optimal.

### 1.3 Summary of study aims

The current study investigated the profile of language abilities and impairments in a sample of high achieving adults with a prior diagnosis of a developmental disorder, with a particular focus on dyslexic adults. We recruited a large sample of adults with a range of developmental disorders, and used a comprehensive language assessment battery that covered a broad range of language skills, including phonological, syntactic, comprehension and narrative skills. Such a wide range of measures was chosen to reflect the range of language problems that have been demonstrated in children with different developmental disorders, including dyslexia, dyspraxia and autism. These language measures were collected for three main aims:

1. **Aim 1:** To provide a characterization of the profile of impairments demonstrated by a sample of adults with developmental disorders across a range of standardized tests, considering similarities and differences between dyslexia and other developmental disorders.
2. **Aim 2:** To investigate differences between dyslexic and non‐disordered groups across measures of reading, oral language and memory/processing.
3. **Aim 3:** To explore whether a data‐driven clustering analysis with these language measures would reveal distinct clusters of profiles of impairment, corresponding to different diagnostic categories.

## 2 METHODS

### 2.1 Participants

Participants were 97 adults (mean age = 24.13, 32 male), 65 of whom had a prior diagnosis of a developmental disorder (developmental disorder group) and 32 of whom had no such diagnosis (non‐disordered group). The two groups did not significantly differ in age (*t*[76.9] = 0.487, *p* = .628). According to self‐reports of handedness, the developmental disorder group consisted of 56 right handers, 8 left handers and 1 ambidextrous; and the non‐disordered group consisted of 30 right handers and 2 left handers. Exclusion criteria for both groups were significant hearing loss, history of neurological disease, head injury or epilepsy. All participants were native speakers of English and had normal or corrected to normal vision. Inclusion in the developmental disorder group did not rely on the application of psychometric criteria from collected data, but simply on self‐report from the participant of a formal diagnosis of a recognized developmental disorder (including dyslexia, dyspraxia and autism) or specific learning difficulties (SpLD). Most participants were current students enrolled on a university or college of higher education course (82 participants); all participants had been through higher education. This was therefore a high achieving sample. Recruitment was predominantly via disability services at local institutes of higher education for the developmental disorder group, and via an online participant recruitment system for the non‐disordered group.

The developmental disorder group consisted of individuals with a range of diagnoses: 40 dyslexia, 10 dyspraxia, 9 dyslexia and dyspraxia, 4 autism and 2 specific learning difficulties (SpLD). Individuals in this latter category did not reach criteria for any specific diagnosis such as dyslexia, but had been diagnosed by their university disability advisory service as having specific processing difficulties relative to other aspects of cognition, such as slower audio‐visual processing.

According to self‐report questionnaire data from the 65 individuals in the developmental disorder group, 50.8% of this sample had received special help or learning support while at school or university. Only 9.2% of the developmental disorder group reported having received speech and language therapy. A family history of similar problems was reported in just over half this group, with 52.3% reporting they had a family member with a history of speech, language, reading or communication disorder. The majority of the participants received their diagnosis while at school (64.6%), with the rest being diagnosed at university (except for one participant diagnosed through work).

Ethical approval for this study was granted by the Research Ethics Committee at the University of Oxford (approval number R40410/RE001). The experiment was undertaken with the understanding and written consent of each participant.

### 2.2 Procedure

Data was collected as part of a larger study of language lateralization (Bradshaw, Woodhead, Thompson, & Bishop, 2020), for which all participants completed an initial session involving administration of a language assessment battery. This session lasted up to 2 h. First, participants filled out a custom‐created questionnaire asking for demographic information, and information on support received at school, history of speech and language therapy, and family history of speech, reading or communication disorders. Three measures of handedness were administered; the Quantification of Hand Preference (QHP) task (Bishop, Ross, Daniels, & Bright, 1996); the Annett peg moving task (Annett, 1985); and the Edinburgh handedness inventory (Oldfield, 1971). A language assessment battery was then administered, consisting of a range of standardized tests designed to tap different aspects of language functioning. These are detailed below. A measure of non‐verbal ability was also obtained using tests one and two of the Cattell Culture fair test (Cattell & Cattell, 1973). Although standardization of this test is old, it was selected because it provides a quick and well‐validated assessment of ability that can be completed by participants independently, and we planned to use raw scores only.

Data collected using these measures was stored on Research Electronic Data Capture (REDCap) electronic data capture tools (Harris et al., 2009) hosted at XXX University. REDCap is a secure, web‐based application designed to support data capture for research studies, providing: (1) an intuitive interface for validated data entry; (2) audit trails for tracking data manipulation and export procedures; (3) automated export procedures for seamless data downloads to common statistical packages; and (4) procedures for importing data from external sources.

### 2.3 Language assessment battery

The tests and their associated measures used for the current study are listed in Table 1, along with the domain that each aims to test. These gave a total of 14 measures (plus a combined score for TOWRE word and non‐word reading used for comparison with test norms). Each test is described in full in the following sections.

**TABLE 1:** Summary of measures from tests administered within the language assessment battery

Instrument	Measure(s)	Domain being tested
Expression, Reception and Recall of Narrative Instrument (ERNNI) (Bishop, 2004)	Mean length of utterance (MLU) Story comprehension	Expressive language and story comprehension
Wechsler Adult Intelligence Scale (WAIS) (Wechsler, 1997)	Digit span overall score	Working memory
Wechsler Abbreviated Scale of Intelligence (WASI) (Wechsler, 1999)	Vocabulary	Verbal IQ
York Adult Assessment Battery‐ Revised (YAA‐R) (Warmington, Stothard, & Snowling, 2013)	Rapid naming objects	Speed of processing
Test of Word Reading Efficiency (TOWRE) (Torgesen, Wagner, & Rashotte, 1999)	Word reading standard score Non‐word reading standard score Overall reading standard score	Single word recognition Phonological skills (decoding)
NEPSY‐ A developmental neuropsychological assessment (Korkman, Kirk, & Kemp, 1998)	Oromotor sequences Non‐word repetition	Articulation/oromotor skills Phonological skills/auditory short term memory
Test of Adolescent and Adult Language (TOAL‐4) (Hammill, Brown, Larson, & Wiederholt, 2007)	Sentence combining	Syntax/sentence production
Communication Checklist self‐report (Bishop, Whitehouse, & Sharp, 2009)	Language structure Pragmatics Social engagement	Communication abilities (oral language skills, pragmatic skills and social engagement)
Cattell Culture Fair Test (Cattell & Cattell, 1973).	Tests 1 and 2 overall score	Non‐verbal intelligence

#### 2.3.1 Expression, reception and recall of narrative instrument (ERRNI)

The ERRNI (Bishop, 2004) is a standardized test in which the participant looks through a set of pictures that tell a story (Fish story) and then narrates that story using the pictures. After an interval of between 15 and 30 min, the participant is asked to retell the story without the pictures. Initial storytelling and story recall are scored for content, and a combined measure of mean length of utterance calculated across the narratives. The participant is also asked some comprehension questions about the story, which tested both factual memory (e.g., What type of pet did the boy have?) and inferences about the mental state of characters (e.g., How did the boy feel when he found the doll?), to give a comprehension score. Raw scores were used for the main analyses, with standard scores used for comparisons with the normative sample (39 UK adults aged 17 to 64 years) (see Section 3).

#### 2.3.2 Wechsler adult intelligence scale (WAIS) IV: Digit span

The WAIS‐IV digit span test (Wechsler, 2008) requires the participant to listen to and report back to the experimenter lists of numbers of increasing length. For the first set of lists, the participants recalls the digits in the same order as given (forwards digit span) to test phonological working memory. For a second set of lists, the participant is instructed to report digits in reverse order (backwards digit span) to test auditory working memory. Scores for backwards and forwards span were combined to give an overall digit span score. Although raw scores were used for most analyses, again standard scores based on the normative sample provided by the test were used for comparisons with the current sample. The WAIS‐IV's norms were derived from a representative sample of 2,200 US adults aged 16 to 90 years. The working memory composite made up of several sub‐tests within the WAIS‐IV including the digit span task is reported by the manual to have reliability of .94.

#### 2.3.3 Wechsler abbreviated scale of intelligence (WASI): Vocabulary

The Vocabulary sub‐test of the WASI (Wechsler, 1999) was administered to obtain a measure of vocabulary knowledge. This requires participants to verbally describe and define words of increasing difficulty/obscurity, which are then scored for accuracy. Standardized age‐normed scores (T‐scores) were calculated using the test manual. These standard scores are based on a normative sample of 2,245 US children and adults aged 6 to 89 years. The reliability of this sub‐test was reported to be high within the adult age‐band (aged 17 to 89 years) of this normative sample, with split‐half reliability of .94 and test–retest reliability of .90.

#### 2.3.4 York adult assessment battery‐ revised (YAA‐R): Rapid naming

The rapid naming sub‐tests of the YAA‐R (Warmington et al., 2013) were administered to assess naming speed/speed of processing. Participants were required to name as fast as possible a series of written digits, and subsequently a series of pictures of objects. Naming rate (items per second) was calculated for digits and objects separately; only the latter was used for the present study. Raw scores were used for all analyses, but data on a normative sample provided by the test were used for comparison with the current sample. This normative sample comprised 106 UK university students aged 18 to 36 years, with no reported history of reading problems. The YAA‐R does not provide information on test–retest reliability for this sub‐test.

#### 2.3.5 Test of word reading efficiency (TOWRE)

The TOWRE (Torgesen et al., 1999) was administered to obtain measures of word and non‐word reading ability. Participants read a list of words or non‐words as fast as they can within a time limit of 45 s. Standardized age‐normed scores were calculated for word and non‐word reading separately, and combined to form an overall standard score. These standard scores provided by the test are based on a normative sample of 1,507 US participants aged 6 to 24 years. For the current analyses, a small adjustment to the upper end of the distribution of these standardized scores was made so as to reduce ceiling effects. High internal reliability was reported for the 18–24 age‐band within this normative sample, with correlations between scores from alternative forms ranging from .89 to .94 across different measures. The test also reports high test‐rest reliability within a sample aged 19 years, with correlations between scores taken at two time points ranging from .82 to .96 across the different measures.

#### 2.3.6 NEPSY: A developmental neuropsychological assessment

Two sub‐tests of the NEPSY (Korkman et al., 1998) were administered; oromotor sequences and non‐word repetition. The oromotor sequences test requires participants to repeat each of a set of phrases or sentences out loud five times in a row; the number of failed attempts was recorded (e.g., omissions, distortions, substitutions of words) and an overall score calculated for the number of successful utterances. The items are selected to be “tongue twisters” and the test is intended to provide a measure of oromotor language skills, but later items also involve a verbal memory component (e.g., repeating “put the pepper beads in the paper bag”). The non‐word repetition task required participants to repeat out loud auditorily presented non‐words. Accuracy of pronunciation of individual syllables was scored to give an overall accuracy score across words. This provides a measure of phonological short‐term memory. Our main analysis used raw scores. The standard scores provided by the manual for these tasks are only for a child sample, but Barry, Yasin, and Bishop (2007) reported data using these tasks with a sample of 33 UK adults; the mean and standard deviation of the scores of this group were used to derive norms for comparison with the current sample. Based on the child sample reported on by the test (aged 3 to 12 years), moderate levels of test‐rest reliability were reported of .67 for the non‐word repetition sub‐test, and 73% consistency of classification for the oromotor sequences sub‐test.

#### 2.3.7 Test of adolescent and adult language (TOAL‐4): Sentence assembly

The sentence assembly sub‐test of the TOAL (Hammill et al., 2007) was administered to obtain a measure of syntactic abilities. The participant was given sets of short sentences written on cards, and required to orally combine each set into a single sentence that preserved the meaning of the set of sentences. This task was self‐paced, and participants were able to view the written sentences throughout. Produced sentences were marked for their grammaticality and content, with full marks awarded for a well‐formed sentence that included all ideas expressed in the set of sentences. Standard scores are provided by the test manual based on a normative sample of 1,671 US participants aged 12 to 24 years. Raw scores were used for the main analyses, but standard scores used to compare the developmental disorder group to this normative group. The TOAL reports a high level of reliability for this sub‐test, with internal consistency reliability of .89 (Cronbach's alpha) averaged across age bands, and a test‐rest reliability coefficient of .93 for the 15‐ to 18‐year age band.

#### 2.3.8 Communication checklist self‐report (CC‐SR)

The CC‐SR (Bishop et al., 2009) provides measures of a person's self‐reported language and communication skills. This is a questionnaire made up of a set of statements for which the participant must report how often each applies to them using a five‐point scale. These statements form three sub‐scales of language structure (oral language ability), pragmatic skills and social engagement. Age‐adjusted standard scores were calculated using the automated scorer provided with the test. These standard scores are based on a normative sample of 481 UK participants between the ages of 10 and 89. The average internal reliability coefficient averaged across the three sub‐scales was reported as .93 with this normative sample.

### 2.4 Data analysis

All analyses were conducted using R software (R Core Team, 2019). An R markdown script that runs all analyses reported here can be found on the OSF page for this project (https://osf.io/bwsha/). The following sections will describe statistical analyses and report results for each of the three study aims in turn.

## 3 AIM 1: CHARACTERIZATION OF THE PROFILE OF IMPAIRMENTS IN A SAMPLE OF ADULTS WITH DEVELOPMENTAL DISORDERS

### 3.1 Analyses

The first aim was to investigate the extent and nature of language impairments present in the developmental disorder group relative to the non‐disordered group. To consider differences between broad diagnostic categories, the developmental disorder group was further decomposed into participants with a dyslexia diagnosis (*n* = 49), and participants with other diagnoses (“non‐dyslexic developmental disorder,” *n* = 16). This latter group was mainly composed of individuals with a dyspraxia diagnosis (*n* = 10), plus four individuals with autism and two with SpLD. Due to the limitations on statistical power imposed by the relatively small sample size in this non‐dyslexic developmental disorder group, mean scores across measures in the three groups are simply presented and discussed qualitatively, without quantitative statistical analyses. In addition, standardized scores were used to compare the performance of each group to that of the normative samples provided by the tests. This was done to demonstrate the extent to which the scores of the developmental disorder groups were in the impaired range relative to general population norms. The percentage of participants scoring below a specified threshold on each measure was calculated for dyslexic, non‐dyslexic developmental disorder and non‐disordered groups separately. A summary of the scores, normative samples and cut‐offs used for each measure is given in Table 2. Where standard scores were provided by the test on an appropriate normative sample, the percentage of participants with a standard score that was one standard deviation below that of the normative sample mean was calculated for each measure. Based on the normal distribution, we would expect around 16% of individuals in the general population to score in this range. Otherwise, alternative cut‐offs were defined as specified in the table.

**TABLE 2:** Summary of procedure for normative comparisons. Details of standardization procedures for comparison of dyslexic, non‐dyslexic developmental disorder and non‐disordered groups with normative samples across the language measures

Measure	Scores used	Normative sample description	Cut‐offs used
ERRNI comprehension	Standard scores	39 UK adults aged 17 to 64 years. Mean score of 102.12 (*SD* = 16.09)	1 SD below normative sample mean
ERRNI mean length of utterance	Standard scores	39 UK adults aged 17 to 64 years, mean score of 98.02 (*SD* = 16.93)	1 SD below normative sample mean
WAIS‐IV digit span	Standard scores	2,200 US adults aged 16 to 90 years.	Score of 7 or below (described as “below average” by Sattler & Dumont, 2004)
WASI vocabulary	T‐scores	2,245 US children and adults aged 6 to 89 years. Mean score of 50 (*SD* = 10).	1 SD below normative sample mean
YAA‐R object rapid naming	Raw scores	106 UK university students aged 18 to 36 years. Mean score of 1.88 (*SD* = .35)	1 SD below normative sample mean
TOWRE overall	Standard scores	1,507 US adults aged 12 to 24 years. Mean score of 100 (*SD* = 15)	1 SD below normative sample mean
NEPSY oromotor sequences	Raw scores	33 UK adults aged 34 to 56 years (from Barry et al., 2007). Mean score of 64 (*SD* = 4.6)	1 SD below normative sample mean
NEPSY non‐word repetition	Raw scores	33 UK adults aged 34 to 56 years (from Barry et al., 2007). Mean score of 41.0 (*SD* = 3.9)	1 SD below normative sample mean
TOAL sentence assembly	Standard scores	1,671 US participants aged 12–24 years. Mean score of 10 (*SD* = 3)	1 SD below normative sample mean
CC‐SR language structure	Scaled scores	481 UK participants aged 10 to 89 years. Mean score of 10 (*SD* = 3)	1 SD below normative sample mean
CC‐SR pragmatics	Scaled scores	As above	1 SD below normative sample mean
CC‐SR social engagement	Scaled scores	As above	1 SD below normative sample mean

### 3.2 RESULTS

Mean scores for the dyslexic, non‐dyslexic developmental disorder and non‐disordered groups are given in Table 3. Visual inspection of the group means suggests similar levels of impairment in dyslexic and non‐dyslexic developmental disorder groups on measures of word reading (TOWRE words), working memory (digit span) and processing speed (YAA‐R rapid naming). The non‐dyslexic developmental disorder group further appears to show particularly low scores for measures of oral language ability, such as ERRNI MLU and CC‐SR language structure. Low scores for this group on the other CC‐SR measures (pragmatic skills and social engagement) reflect the presence of individuals with a diagnosis of autism in this group. The distribution of data and correlation matrices across the 14 measures for each of the three groups can be found in the supplementary material for this paper (Data S1).

**TABLE 3:** Mean (and standard deviation) scores in the dyslexic, non‐dyslexic developmental disorder and non‐disordered groups

Measure	Mean (SD) non‐disordered cases (*N* = 32)	Mean (SD) dyslexics (*N* = 49)	Mean (SD) non‐dysl dev. Disorder (*N* = 16)
Literacy			
TOWRE words	105.38 (10.81)	91.61 (13.59)	96.63 (16.46)
Phonological processing			
TOWRE non‐words	109.81 (10.59)	92.57 (13.46)	106.88 (12.02)
NEPSY non‐word repetition	42.47 (2.96)	40.49 (4.44)	41.63 (2.36)
Processing skills			
Digit span total score	19.97 (3.04)	16.82 (3.86)	17.88 (3.93)
YAA‐R objects rapid naming rate	2.06 (0.24)	1.75 (0.37)	1.77 (0.27)
Structural oral language skills			
ERRNI mean length of utterance	12.01 (1.73)	11.04 (2.12)	10.21 (1.45)
NEPSY oromotor sequences	63.53 (3.75)	61.10 (5.44)	63.44 (3.33)
CC‐SR language	10.28 (3.93)	8.63 (3.73)	7.88 (1.75)
Vocabulary and comprehension			
WASI vocabulary	62.88 (6.63)	61.84 (7.24)	62.81 (5.02)
ERRNI comprehension	14.88 (1.86)	14.27 (1.98)	14.63 (1.86)
Syntactic processing			
TOAL sentence assembly	14.88 (2.92)	13.94 (3.48)	14.69 (2.87)
Social and pragmatic skills			
CC‐SR pragmatics	10.75 (2.88)	10.16 (3.73)	7.63 (2.78)
CC‐SR social	11.75 (2.87)	11.04 (3.14)	7.13 (4.35)
General intelligence			
Cattell non‐verbal ability	19.88 (2.86)	20.16 (2.17)	19.88 (3.36)

The percentages of participants in each of these three groups scoring below cut‐offs derived from normative samples (see Table 2) are given in Table 4. These demonstrate that across measures, a low to moderate proportion of participants within the dyslexic and non‐dyslexic developmental disorder groups are impaired relative to the general population; in particular, relatively high percentages are seen for YAA‐R rapid naming, TOWRE word and non‐word reading, NEPSY oromotor sequences, TOAL sentence assembly and CCSR language structure measures. For the non‐disordered group, percentages are generally very low, with notable exceptions being TOAL sentence assembly, CC‐SR language structure and NEPSY oromotor sequences. This could be attributable to demographic differences between this sample and the normative samples employed by the tests, for example in age and nationality. Comparison of dyslexic and non‐dyslexic developmental disorder groups revealed that, although the non‐dyslexic developmental disorder group was generally less impaired than the dyslexic group, there were nevertheless a number of measures for which the non‐dyslexic group contains a significant number of individuals in the impaired range. Particularly striking is the relatively high percentage (25%) of non‐dyslexic developmental disorder participants scoring below normative levels for YAA‐R rapid naming, compared to 0% in the non‐disordered group. This is consistent with their low mean score for this measure.

**TABLE 4:** Comparison of developmental disorder and non‐disordered groups with normative samples. Percentages of dyslexic, non‐dyslexic developmental disorder and non‐disordered groups scoring below normative cut‐offs across the 12 language measures

Measure	Percentage below		
	Non‐disordered group (*N* = 32)	Dyslexic (*N* = 49)	Non‐dyslexic dev. disorder (*N* = 16)
ERRNI comprehension	3.13	6.12	6.25
ERRNI MLU	0	0	6.25
Digit span	0	10.20	6.25
WASI vocabulary	0	0	0
YAA‐R objects rapid naming rate	0	32.65	25
TOWRE overall	0	38.78	12.5
NEPSY oromotor sequences	15.63	28.57	18.75
NEPSY non‐word repetition	3.13	16.33	6.25
TOAL sentence assembly	31.25	36.73	25
CC‐SR language structure	21.88	36.73	18.75
CC‐SR pragmatics	6.25	12.24	25
CC‐SR social engagement	3.13	12.24	50

## 4 AIM 2: COMPARING DYSLEXIC AND NON‐DISORDERED GROUPS

### 4.1 Statistical analyses

To investigate differences between the dyslexic and non‐disordered groups more specifically, we used a two‐step approach to determine which indicators showed a statistically significant difference. Firstly, we obtained effect sizes (Cohen's d) for differences between dyslexic and non‐disordered participants for each measure, using either parametric t‐tests or non‐parametric Mann–Whitney‐U tests, depending on the normality of each measure within each group. In the non‐parametric case, Cohen's d was calculated from the *r* effect size estimate using an online converter (https://www.psychometrica.de/effect_size.html). This step's purpose was solely to establish effect sizes for comparison with those reported in previous literature, rather than to determine statistical significance of differences.

In a second step, we used a multivariate test to establish whether there was evidence for a statistically significant difference between groups, and a post‐hoc approach using bootstrapped confidence intervals to determine which measures specifically showed significant differences. Despite the application of multiple univariate tests being an established approach in previous literature, this is suboptimal as it results in inflated type one error; a Bonferroni correction, however, is too stringent, given that the tests are correlated. Therefore, this analysis was followed up using a non‐parametric multivariate test (Nordhausen & Oja, 2015). Use of a non‐parametric test was necessary due to the non‐normality of some of the language measures. This test can be considered as a multivariate extension of the Wilcoxon‐Mann–Whitney test, but with altered calculation of signs and ranks to reflect the multidimensionality of the data. The test statistic, *Q* ^*2*^, follows a chi‐squared distribution and thus can be compared with the chi‐squared critical value to test for significance. This non‐parametric multivariate test was run using the R package MNM (Nordhausen & Oja, 2015) to compare performance of the two groups (dyslexic versus non‐disordered) on the 14 measures. For this analysis, bootstrapping (Efron, 1979) was used to obtain 95% confidence intervals for mean differences between groups on each of the measures, in order to ascertain on which measures the two groups significantly differed, while controlling the type 1 error rate.

In line with previous research, it was predicted that the two groups would differ on measures of word reading, phonological skills (non‐word reading, non‐word repetition), phonological short term memory/working memory (digit span), speed of processing (rapid naming), structural oral language (oromotor sequences, CCSR language structure, ERRNI MLU) and sentence assembly (TOAL). In contrast, it was predicted that the two groups would not differ on measures of vocabulary (WASI), comprehension (ERRNI comprehension) and non‐verbal ability (Cattell).

### 4.2 RESULTS

**FIGURE 2:**
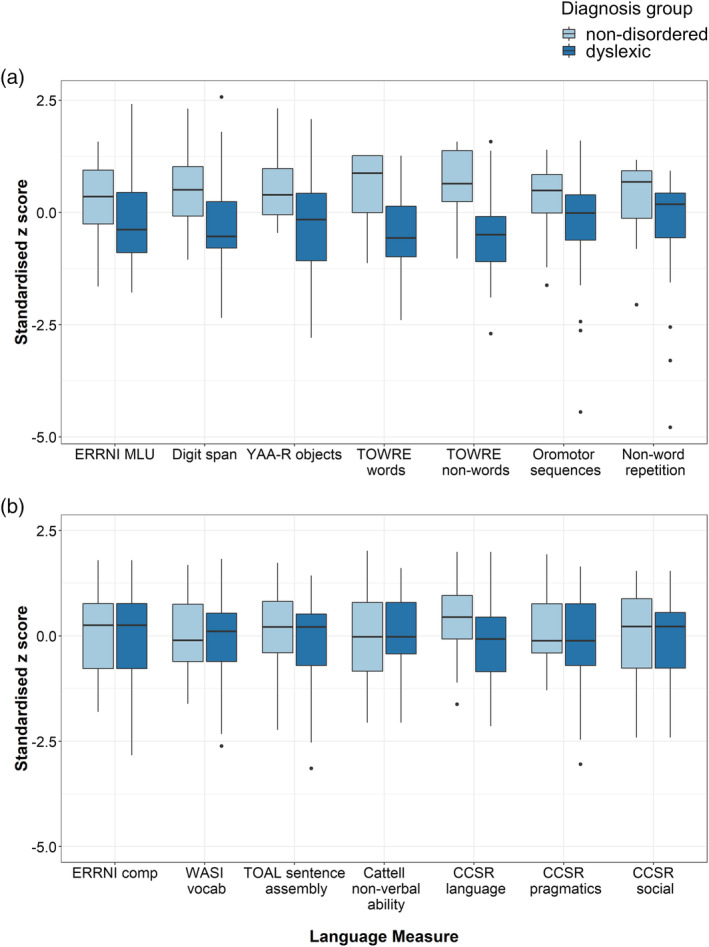
Standardized scores in the dyslexic and non‐disordered groups. Boxplots of standardized z scores (calculated based on the mean and standard deviation of the whole sample) for each of the 14 language measures in the dyslexic and non‐disordered groups. Dark lines indicate medians for each group, dots indicate outliers [Colour figure can be viewed at wileyonlinelibrary.com]

Standardized scores for dyslexic and non‐disordered groups across the 14 measures are given in Figure 2. Effect sizes for differences between these groups are given for the full set of language measures in Table 5. Non‐parametric multivariate analysis showed that the two groups differed across the measures as a set, *Q* ^2^ = 36.29, *df* = 12, *p* < .001. Bootstrapped 95% confidence intervals revealed significant differences between groups on TOWRE word and non‐word reading, YAA‐R object rapid naming, digit span, NEPSY oromotor sequences and non‐word repetition. No significant differences were found for Cattell non‐verbal ability, WASI vocabulary, ERRNI comprehension, ERRNI MLU, TOAL sentence assembly, CC‐SR language structure, CC‐SR pragmatic skills or CC‐SR social engagement. A plot of these bootstrapped 95% confidence intervals can be found in the R markdown document for these analyses which is available on OSF (https://osf.io/bwsha/); significant differences were indicated by confidence intervals that did not overlap with zero.

**TABLE 5:** Comparison of dyslexic and non‐disordered groups. Table gives effect sizes (Cohen's d) based on univariate tests and significance based on multivariate bootstrapped 95% confidence intervals (CIs) for differences between dyslexic and non‐disordered participants. The sign of effect sizes has been inverted such that lower performance in dyslexics versus non‐disordered cases corresponds to a positive effect size. Stars indicate that group differences were significant at the *p* < .05 level (i.e., the bootstrapped 95% confidence intervals from the multivariate test did not overlap with zero). Measures that were non‐normal whose effect sizes were obtained using non‐parametric univariate tests are marked with ^1^

Measure	Effect size (d)	Significance (multivariate CIs)
Literacy		
TOWRE words^1^	1.115	*
Phonological processing		
TOWRE non‐words^1^	1.469	*
NEPSY non‐word repetition^1^	0.588	*
Processing skills		
Digit span total score	0.886	*
YAA‐R objects rapid naming rate	0.956	*
Oral language skills		
ERRNI mean length of utterance^1^	0.597	
NEPSY oromotor sequences^1^	0.509	*
CC‐SR language^1^	0.434	
Vocabulary and comprehension		
WASI vocabulary	0.148	
ERRNI comprehension	0.315	
Syntactic processing		
TOAL sentence assembly^1^	0.207	
Social and pragmatic skills		
CC‐SR pragmatics	0.171	
CC‐SR social^1^	0.211	
General intelligence		
Cattell non‐verbal ability	−0.117	

## 5 AIM 3: CORRESPONDENCE BETWEEN DIAGNOSTIC LABELS AND DATA‐DRIVEN SUB‐GROUPS OF PARTICIPANTS

### 5.1 Statistical analyses

The aim of this analysis was to derive data‐driven sub‐groups from the language measures, and to examine whether these corresponded to diagnostic labels. To obtain data‐driven sub‐groups, an unsupervised machine learning (UML) approach was used on all 14 language measures. Interpretation of clusters can become difficult when including too many input variables for clustering, known as “the curse of dimensionality” (Hastie, Tibshirani, & Friedman, 2001); despite this, 14 variables was considered an appropriate number for this sample size and not sufficiently large to warrant concern. Scores on these 14 measures were converted to standardized *z*‐scores based on the mean and standard deviation of the whole sample for this analysis.

The UML approach seeks to find structure in unlabelled input data; that is, the groups are not pre‐specified but derived by looking at similarities between cases in order to define homogeneous sub‐groups. A *k*‐means clustering analysis was used to group individuals or “observations” (Hartigan & Wong, 1979). The aim of *k*‐means clustering is to partition observations into a fixed number of *k* homogenous clusters. Each observation is considered as a point in multi‐dimensional space. Initially, *k* number of points in this space are chosen to represent the centre of clusters, and observations are assigned to their nearest cluster. Cluster centres are then re‐calculated based on their cluster members, and observations reassigned to clusters based on these new centres. This process is repeated iteratively until cluster memberships' stabilize. This was implemented using the R function “kmeans” within the package “stats” (R Core Team, 2019), which selects the most optimal partitioning of cases based on which yields the smallest total within‐cluster sum of squares.

Three techniques were used in parallel to determine the number of clusters (*k*). Firstly, a scree plot was constructed by iteratively fitting the *k*‐means clustering process over different values of *k* and calculating the average within‐cluster sum of squares, which were then plotted against each other. Typically, the optimal value of *k* is chosen by identifying the “elbow” within the scree plot. Secondly, we constructed a silhouette score plot. This provides a “cohesion” score (how similar an observation is to its own cluster). Clusters with higher cohesion scores are interpreted as showing a better overall fit. Finally, the gap statistic was calculated which compared the total intra‐cluster variation for different values of *k* against their expected values under a null distribution (i.e., a reference distribution that is standard to the test statistic to gauge statistical significance; see Tibshirani, Walther, & Hastie, 2001). The optimal solution looks for the number of clusters that maximizes the gap statistic.

Cluster models were compared using three fit indices: compactness, the Dunn index and the Rand Index. The *k*‐means algorithm seeks to minimize the sum of squares of the observations in relation to their particular cluster centroid. The compactness of the clustering can be presented as a percentage derived from the ratio of the between sum of squares and the total sum of squares, which gives an indication of how close the points are to a cluster centroid (i.e., how similar the points are in the same cluster). The Dunn index is another measure of the internal clustering validation. It takes into account both the distance from points to their cluster centroid, and the distance between clusters. A larger Dunn index is optimal. The Rand index is a measure of the similarity of two data clusterings, and was used here to determine the level of agreement between groupings based on pre‐known diagnosis categories and groupings in the clustering solution yielded by the UML analysis. This index can thus be used to test how well the data‐driven groupings match up to the diagnostic labels. This index has a range from −1 (no agreement) to +1 (perfect agreement).

### 5.2 RESULTS

**FIGURE 3:**
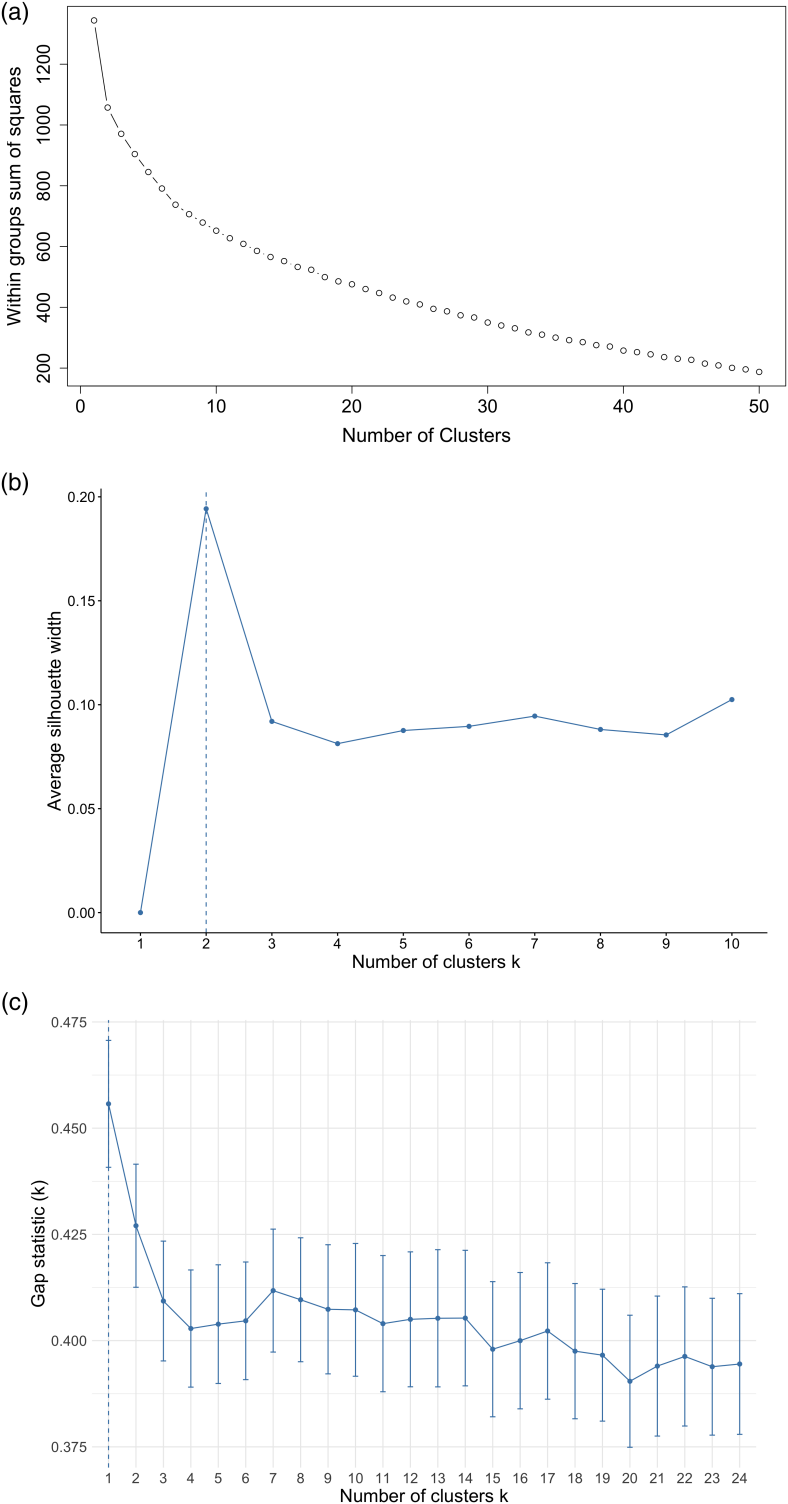
Criteria for determining number of clusters in UML analysis. (a) Scree plot showing within‐cluster sum of squares calculated across a range of values of *k* (number of clusters). (b) Silhouette score plot showing cohesion scores against a range of values of *k*. (c) Plot of the Gap statistic (based on intra‐cluster variation) against a range of values of *k* [Colour figure can be viewed at wileyonlinelibrary.com]

**FIGURE 4:**
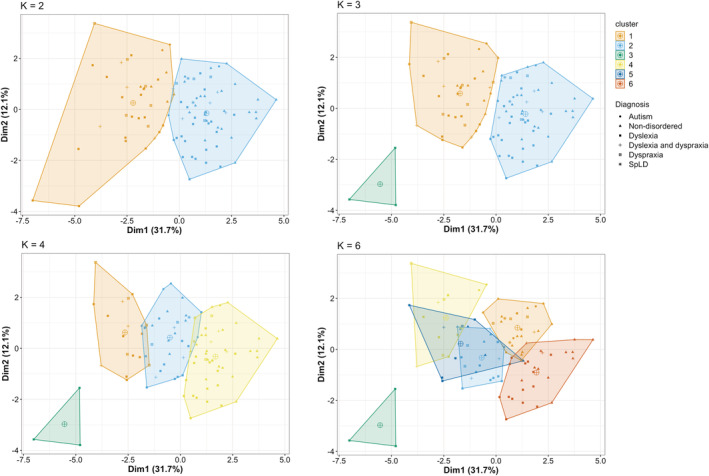
Grid of cluster plots. Scatter plot showing points relative positions of observations (participants) on the first two discriminant functions for each *k*‐means model (*k* = 2, 3, 4, 6). Large circles with a cross indicate the centroid (central point) of each cluster. SpLD, specific learning difficulties [Colour figure can be viewed at wileyonlinelibrary.com]

Firstly, we considered how many clusters should be included in the clustering model. The scree plot for the data (Figure 3a) was found to show a relatively smooth curve, with no clear indication of an elbow inflection point. We then considered two additional approaches: the silhouette scores plot and the gap statistic (Figure 3b,c). Inspection of the silhouette scores plot revealed that the optimal solution appeared to be a two‐cluster grouping. The gap statistic however was not in agreement with this, indicating that a one‐factor solution was best. Based on this conflicting evidence of the optimal number of clusters and given the number of diagnosis groups, we compared four different *k*‐cluster solutions (*k* = 2, 3, 4, 6) to see which provided the best fit to the data. Figure 4 presents the cluster plots for each *k*‐means model (plotting the first two discriminant functions).

It can be seen in Figure 4 that each cluster model (at different values of *k*) produces clusters that are relatively distinct, with centroids that are well spaced from each other (central points of each cluster as indicated by larger symbols on Figure 4). Table 6 presents the fit indices for the four cluster models so that relative performance can be assessed. The statistics indicate a mixed picture with no real consistency in the optimal *k*‐means model. The first compactness statistic indicates how similar members of the cluster are to each other. Across models the compactness is generally low, indicating that no particular model is providing a satisfactory model fit to the data. If higher *k* models were included, we might see a higher compactness, but this would defeat the purpose of trying to determine if a realistic clustering scenario would be comparable to the diagnostic labels in the data.

**TABLE 6:** Comparison of different *k*‐means clustering models

*k* clusters	Compactness statistic	Dunn index	Rand index
**2**	21.3%	.246	.036
**3**	27.8%	.312	.019
**4**	32.7%	.276	.046
**6**	41.2%	.319	.071

The Dunn index (another measure of internal clustering validation) indicated that the six cluster solution was the best among the four models at .319 (although the difference is very small between models). The Rand index also indicated that the six cluster solution provided a cluster allocation that was most similar to the diagnostic labels. It must be highlighted however that all solutions provided a poor correspondence to the diagnostic labels (no model exceeded .071 which is relatively weak evidence of any correspondence).

**FIGURE 5:**
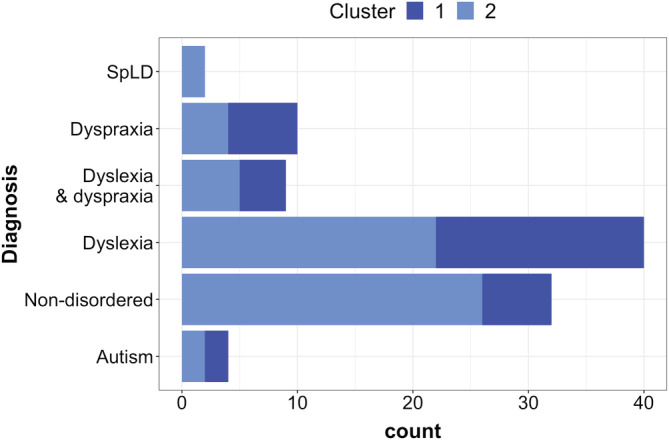
Cluster composition, *k* = 2. Make‐up of the sample grouped into the two clusters by the *k*‐means clustering analysis [Colour figure can be viewed at wileyonlinelibrary.com]

Although there was no clear “winning” model that provided a consistently optimal fit to the data across these measures, to illustrate in more detail one possible cluster solution we now present the two‐cluster model. This model provided the best fit according to the Silhouette score plot. The make‐up of the two clusters derived by this approach in terms of diagnoses of the grouped cases is given in Figure 5. Cluster one contained 36 cases, most of whom had a dyslexia diagnosis (18 dyslexia, four dyslexia plus dyspraxia) with a further 6 with dyspraxia, 2 with autism and 6 with no disorder. Cluster two contained 61 cases, most of whom were cases with no disorder (26), with a selection from the other diagnosis categories (22 with a dyslexia diagnosis, 5 dyslexia plus dyspraxia, 4 dyspraxia, 2 autism and 2 SpLD). Overall therefore, it can be seen that different diagnoses are not clearly separated out across the clusters.

Mean standardized *z*‐scores in the two clusters on the 14 language measures used in the clustering analysis are given in Table 7. As can be seen, cluster one is characterized by poorer performance across the measures compared to cluster two; this suggests that the difference between clusters is simply one of severity, rather than test profile.

**TABLE 7:** Performance of the two clusters. Mean standardized scores (and standard deviation) for the two clusters yielded by the *k*‐means clustering analysis

Measure	Mean (SD) cluster 1 (*N* = 36)	Mean (SD) cluster 2 (*N* = 61)
ERRNI comprehension	−0.40 (1.03)	0.24 (0.90)
ERRNI mean length of utterance	−0.24 (0.96)	0.14 (1.01)
Digit span	−0.63 (0.85)	0.37 (0.89)
WAIS vocabulary	−0.58 (0.99)	0.34 (0.84)
YAA‐R objects rapid naming	−0.70 (0.89)	0.41 (0.82)
TOWRE word reading	−0.74 (0.89)	0.44 (0.78)
TOWRE non‐word reading	−0.62 (0.91)	0.36 (0.87)
NEPSY oromotor sequences	−0.52 (1.24)	0.31 (0.67)
TOAL sentence assembly	−0.45 (1.20)	0.27 (0.75)
Non‐word repetition	−0.51 (1.27)	0.30 (0.63)
Cattell non‐verbal ability	−0.25 (0.99)	0.15 (0.98)
CC‐SR language	−0.82 (0.87)	0.49 (0.81)
CC‐SR pragmatics	−0.73 (0.87)	0.43 (0.82)
CC‐SR social	−0.79 (0.97)	0.47 (0.67)

Overall, the results from this clustering analysis suggest that scores on the 14 language measures included in the current assessment battery did not show a cluster structure that corresponded well to the diagnosis categories present in the sample (indicated by the low Rand indices). Furthermore, none of the models provided a particularly good fit to the data (indicated by the low compactness statistics), suggesting that this data did not show a clearly defined cluster structure.

**FIGURE 6:**
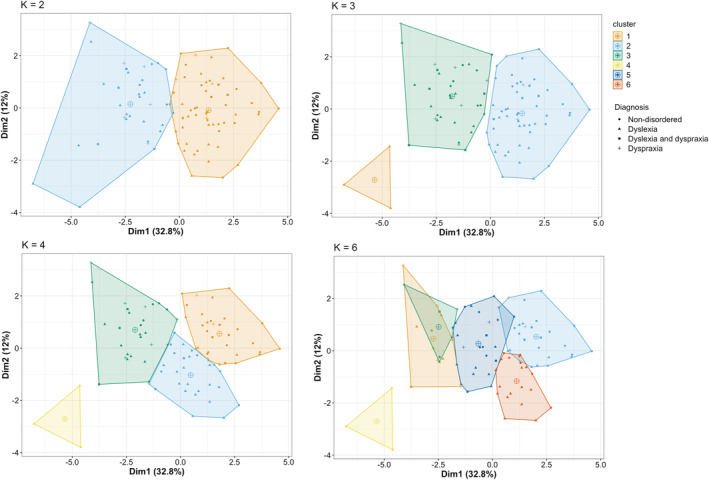
Grid of cluster plots for constrained sample. Scatter plot showing points relative positions of observations (participants) on the first two discriminant functions for each *k*‐means model (*k* = 2, 3, 4, 6), performed on data excluding cases of autism and SpLD. Large circles with a cross indicate the centroid (central point) of each cluster. SpLD, specific learning difficulties [Colour figure can be viewed at wileyonlinelibrary.com]

One possibility is that this reflects the inclusion of very heterogeneous disorders in our sample; in particular, the small number of individuals with a diagnosis of autism or SpLD. It is possible that restricting the analysis to include only non‐disordered participants and cases of dyslexia and dyspraxia (disorders which are known to have overlapping profiles of impairment) would reveal a cluster structure showing a more well‐defined cluster structure and better agreement with groupings based on diagnostic labels. Therefore, we re‐ran this clustering analysis excluding cases with autism and SpLD. Figure 6 shows the cluster structure plots across four values of *k* (plotting the first two discriminant functions). It can be seen that these cluster solutions are very similar to those seen for the full sample with *k* values of 2 and 3 (see Figure 4), with some differences between the two at *k* values of 4 and 6. As can be seen in Table 8, however, this analysis found that values of the compactness statistic and Rand index across the different *k*‐cluster models remained similarly low, indicating that again there was little correspondence between data‐driven clusters and diagnostic labels.

**TABLE 8:** Comparison of different *k*‐means clustering models from the dataset with cases of autism and SpLD removed

*k* clusters	Compactness statistic	Dunn index	Rand index
2	22.6%	.256	.044
3	28.9%	.311	.028
4	34.2%	.279	.105
6	42.7%	.297	.062

## 6 GENERAL DISCUSSION

### 6.1 Summary of findings

This study aimed to examine the profile of impairment in a sample of high achieving adults with a history of developmental disorders. We showed that these adults were impaired relative to non‐disordered participants across a range of tests of language, memory and processing skills. When considering specifically those participants with a diagnosis of dyslexia, impairments were found on measures of single word reading, phonological skills, processing speed, working memory and structural oral language skills. Spared functioning was seen for measures of non‐verbal intelligence and vocabulary, in line with previous research (e.g., Hatcher et al., 2002), as well as for measures of syntactic structure building and story comprehension (non‐reading based). Finally, a data‐driven clustering analysis found that across different model solutions with different numbers of clusters, data‐driven groups did not correspond well with diagnostic labels. Such clusters as emerged were distinguished by severity rather than test profile.

### 6.2 Aim 1: Characterization of the profile of impairments in a sample of adults with developmental disorders

Comparisons of performance across our measures found that in a high achieving adult sample (the majority of whom were university students), a prior diagnosis of a developmental disorder was associated with poorer language and memory skills relative to a comparable sample of university educated adults without such disorders. For both dyslexic and non‐dyslexic developmental disorder groups, similar levels of impairment were found for measures of word reading, working memory and processing speed (rapid naming). In particular, several individuals in the non‐dyslexic developmental disorder group scored below norm cut‐offs for rapid naming, with a very similar mean reading rate to dyslexics. This is consistent with a slower speed of processing in this group, which may underlie their poorer performance for word reading. Furthermore, this group demonstrated signs of poorer oral language skills compared to both non‐disordered cases and dyslexics on measures that relate to everyday use of language (mean length of utterance in free narrative, and a self‐report measure of difficulties with oral language in everyday life). The heterogeneous nature of this group makes it difficult to draw conclusions specific to particular diagnoses; however, given the high number of dyspraxic individuals in this group, this provides some insight into the types of difficulties that may be associated with this disorder in adulthood.

Some caution needs to be exercised however when considering normative cut‐offs for a few of the measures, given some instances of high percentages of non‐disordered participants scoring below cut‐offs; notably on the TOAL sentence assembly task and CC‐SR language structure measure. This highlights the dangers of relying on test norms to identify impairment, especially for tests that were normed on populations that are very different in age, background or geographical location. For a more realistic estimate of impairment profile, we need well‐matched groups of non‐disordered and disordered participants from the same population, as was done in the current study (the majority of participants across these two groups were students of a similar age from the same university).

Bearing that caveat in mind, comparisons with test standardization norms indicated that a substantial proportion of both developmental disorder groups demonstrated skills within the normal range when compared to standardized test norms based on more representative population samples. This confirms that the current sample was indeed a highly functioning sample compared to the general population, and was not severely impaired in terms of everyday functioning. Nevertheless, relative to the well‐matched non‐disordered group, these adults with developmental disorders still show persisting areas of weakness. This indicates that success in entering higher education is not always associated with complete resolution of difficulties experienced in childhood. These individuals thus likely still require additional support in higher education in order to attain a level of academic success that is on par with that of their unaffected peers.

### 6.3 Aim 2: Determination of which measures were most sensitive for discriminating dyslexic from non‐dyslexic participants

Analyses comparing individuals with a diagnosis of dyslexia to non‐disordered cases yielded results that were in good agreement with previous literature. As expected, impairments were demonstrated on measures of phonological skills (non‐word reading and non‐word repetition), rapid naming and working memory. Interestingly, impairments were also demonstrated on measures of structural oral language skills; moderate effect sizes based on univariate tests were found for ERRNI mean length of utterance and the NEPSY oromotor sequences sub‐test (the latter reaching significance in the multivariate test). Lower language structure composite scores on the CC‐SR were also apparent in the dyslexic group, reflected in the moderate percentage (36%) of individuals scoring below norm levels.

The NEPSY oromotor sequences test is defined by the NEPSY manual as assessing “oromotor coordination,” and so can be characterized as a test of articulatory oral language skills. However, it is important to note that this test does also place additional demands on other processes, such as phonological short‐term memory, cognitive monitoring and attention. It is therefore possible that poorer performance on this test could reflect impairments in one or several of these other processes, apart from problems with articulatory oral motor skills.

Impairments in structural language skills have however been reported in some previous studies of adults with dyslexia. A recent study by Wiseheart and Altmann (2018) using a spoken sentence generation task found that dyslexic college students were slower to respond and produced sentences that were less fluent, grammatical and complete. These demonstrations of impairments in structural language skills in high achieving dyslexic students are striking. Together with the results from the current study, this evidence suggests that structural language skills including articulatory skills deserve more attention in future studies of adults with dyslexia.

Conversely, our study found that other aspects of oral language skills were spared in our sample of adults with dyslexia. Consistent with previous research, vocabulary skills were found to be unaffected (Hatcher et al., 2002). This is consistent with the idea that good oral language skills in the form of a strong vocabulary may offer a compensatory or protective factor in dyslexia (Cavalli et al., 2016); this might have enabled these adult participants to successfully enter higher education. Spared performance was also found for a non‐reading based measure of comprehension from a self‐narrated story (ERRNI). Previous research has reported that comprehension from reading can be affected in adults with dyslexia, particularly when under timed conditions (Pedersen, Fusaroli, Lauridsen, & Parrila, 2016). The current finding suggests that general comprehension of material accessed in a manner that does not involve reading is however intact. The finding that groups did not differ on the TOAL sentence assembly sub‐test is somewhat inconsistent with previous research reporting difficulties with syntactic processing in adults with dyslexia (Wiseheart, Altmann, Park, & Lombardino, 2009), but the relatively high rate of impaired scores in the non‐disordered group suggests that norms on this test may be inappropriate for UK students. As expected, social and pragmatic skills as assessed on self‐report measures (CC‐SR) were also unaffected in dyslexic participants.

### 6.4 Aim 3: Correspondence between diagnostic labels and data‐driven sub‐groups of participants

Recently, there have been a number of studies applying unsupervised machine learning approaches to data from samples of children with developmental disorders (Astle et al., 2019; Bathelt et al., 2018). These tend to conclude that diagnostic labels are not good predictors of data‐driven cluster membership. The current clustering analysis provides preliminary evidence that the same pattern may be found with an adult sample. Across multiple models with different numbers of clusters, correspondence between data‐driven clusters and diagnostic groupings was very low (as indicated by the Rand index). This suggests that not all individuals with the same diagnostic label demonstrated the same profile of impairment on our set of language measures.

Across multiple measures of fit, there was no single model (i.e., value of *k*) that provided an optimal fit to the data. This suggests that, based on scores from the current broad battery of language measures, this sample did not show a clearly defined cluster structure. For illustrative purposes, we presented a two‐cluster model to demonstrate how the data‐driven clustering would divide up the sample. Interestingly, this split the sample into a poorly performing group and a typically performing group, with different diagnoses scattered across the two clusters. Thus, in this cluster solution the difference between clusters was simply one of severity, rather than test profile. This has similarities with a clustering analysis presented by Dyck et al. (2011) using a similarly broad battery of measures of language, motor, social and memory skills. In a large sample of children with five different disorders, clustering divided the sample into a better performing and a poorer performing group, again with typical and disordered cases being scattered across both clusters. However, it should be stressed that the two cluster model presented in the current paper was not a “winning” or optimal model, and is presented for illustrative purposes only.

Overall, this analysis highlights how the diagnostic label of an individual does not necessarily predict well the type of support that individual will need; support given should instead be assessed relative to an individual's profile of strengths and weaknesses, rather than in relation to a diagnostic category. However, it should be noted that the current clustering analysis was highly exploratory, and carried out on a relatively small sample; this pattern of findings thus requires replication in a much larger sample, ideally in a pre‐registered study. Such future work may consider focusing on a narrower range of developmental disorders using measures that have high relevance to their defining characteristics; it is possible that this would result in a more clearly defined cluster structure. One interesting example would be to focus on adults with dyslexia and dyspraxia, two disorders that show a high level of comorbidity (Kaplan et al., 1998) and have been shown to share some common areas of impairment in childhood, for example, phonological skills (Dewey, Kaplan, Crawford, & Wilson, 2002). It will be important however, for such work to keep in mind that similarities between cases with different diagnoses at the behavioural level do not necessarily imply shared causes at the cognitive, neurobiological and genetic levels (see Figure 1).

### 6.5 Limitations of the study

There are a number of limitations of the current study which constrain the interpretation of the findings. The failure of the data‐driven clustering analysis to clearly separate out cases of dyslexia (the most frequent developmental disorder in our sample) from other cases may be due to the absence of certain measures more specifically relevant to dyslexia. In particular, measures of phonological awareness, spelling and writing could have increased the sensitivity of the clustering analysis for distinguishing cases of dyslexia from the other categories. Their omission reflects the fact that this test battery was not designed to be specifically tailored to dyslexia, but rather to address a broad range of language functions that may be differentially affected in different disorders. Selection was thus constrained by the need to compile a comprehensive battery that would not be too lengthy to administer.

A further limitation of the study is the small number of participants in diagnosis categories other than dyslexia, with only 10 cases of pure dyspraxia, 4 of autism and 2 of SpLD. This limited our comparisons of the non‐dyslexic developmental disorder group with the other groups to be descriptive rather than statistical. The sample was recruited predominantly through university disability advisory services with the aim of collecting a sample with a broad range of language, literacy and communication skills rather than with regard to specific diagnoses. The over‐representation of dyslexia in the resulting sample was thus unexpected and not reflective of skewed recruitment sources.

Lastly, it should be acknowledged that this sample of adults with developmental disorders is likely to be a highly skewed sample. Recruitment was predominantly via the Disability Services at the University of Oxford, resulting in a sample with a large proportion of high achieving adults with developmental disorders. The findings with this sample may therefore not be representative of samples from other institutes of higher education. It should be further pointed out that adults with a history of learning disabilities who reach higher education are in general likely to be a highly selective sample compared to the general population with learning difficulties. For example, a recent study by Chatzitheochari and Platt (2019) reported that the level of parental education in a sample of adolescents with disabilities who had continued in full‐time upper secondary education was in fact higher than that of their counterparts without disabilities, suggesting a skew towards high SES among these individuals.

## 7 SUMMARY AND CONCLUSIONS

The findings presented in this paper provide insight into the profile of impairments demonstrated by high functioning adults with a history of developmental disorders. This adds to the growing body of evidence suggesting that although these individuals can compensate for their difficulties to a point that enables them to achieve academic success in higher education, they still experience areas of weakness that are likely to put them at a disadvantage during their studies. Difficulties with reading familiar and unfamiliar words as well as limitations in working memory in students with dyslexia would be expected to both hinder learning from written material (inherent to most courses of study) and result in poorer performance under timed exam conditions. This suggests that the common practise of allowing dyslexic participants extra time in exams is justified. In addition to these well‐established difficulties, impairments in structural oral language skills in adults with dyslexia and other related disorders as demonstrated here deserve more attention in future research. The preliminary evidence presented in the current data‐driven clustering analysis with this relatively small sample suggests that the type of support given to an adult student with a developmental disorder should be based on their individual needs, rather than their diagnostic label.

## Supporting information


**Data S1** Supporting information

## Data Availability

The data that support the findings of this study are openly available on Open Science Framework (OSF) at https://osf.io/bwsha/ (DOI: 10.17605/OSF.IO/BWSHA).
