# Supplementary figures and images for: Profile of language abilities in a sample of adults with developmental disorders
Source: Dyslexia. 2020 Nov 17;27(1):3–28. doi: 10.1002/dys.1672 (PMC7894539; doi:10.1002/dys.1672)

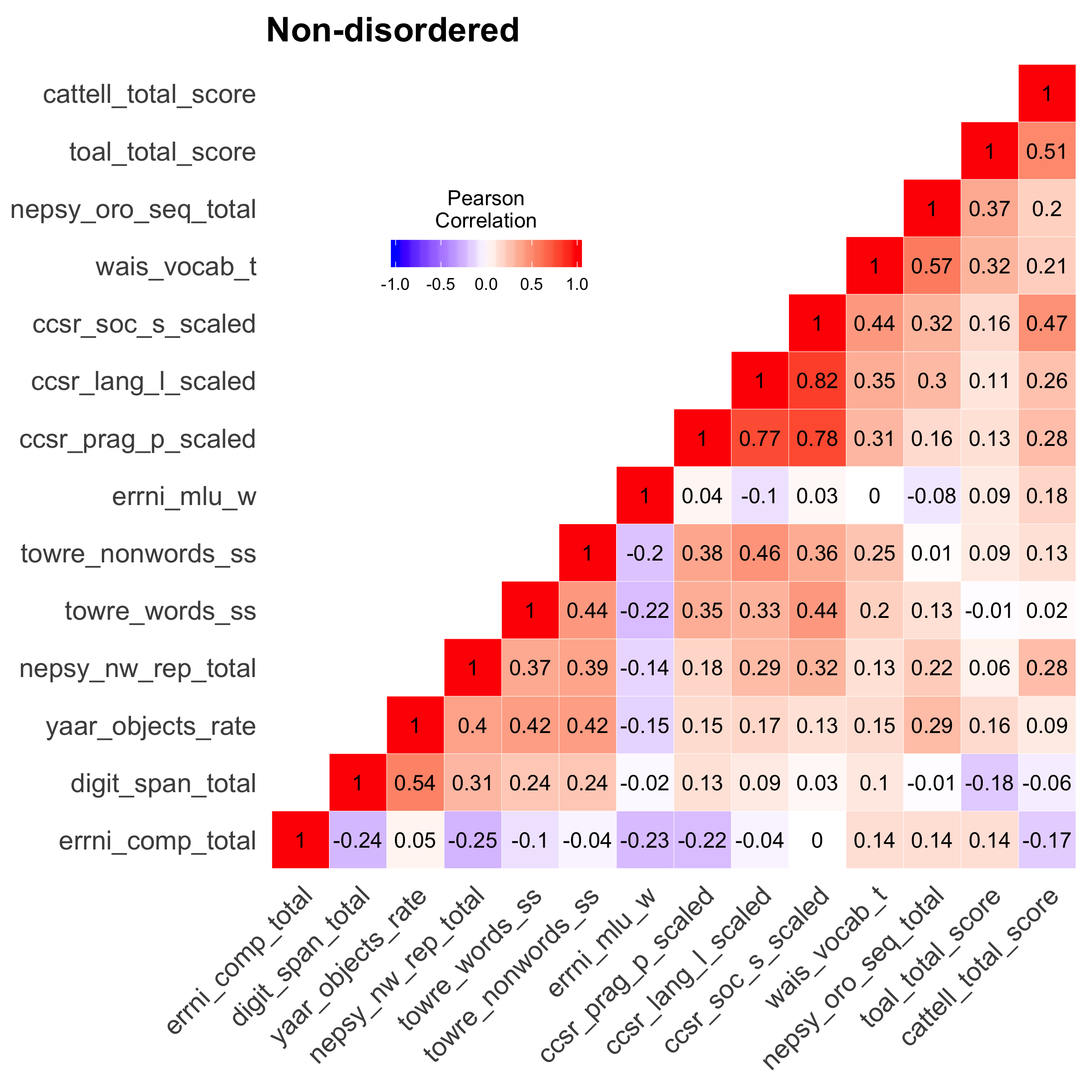

Supplement: Supplementary file 1 — Data S1 Supporting information [file DYS-27-3-s001.zip › DYS_1672_Appendix 2a.png]

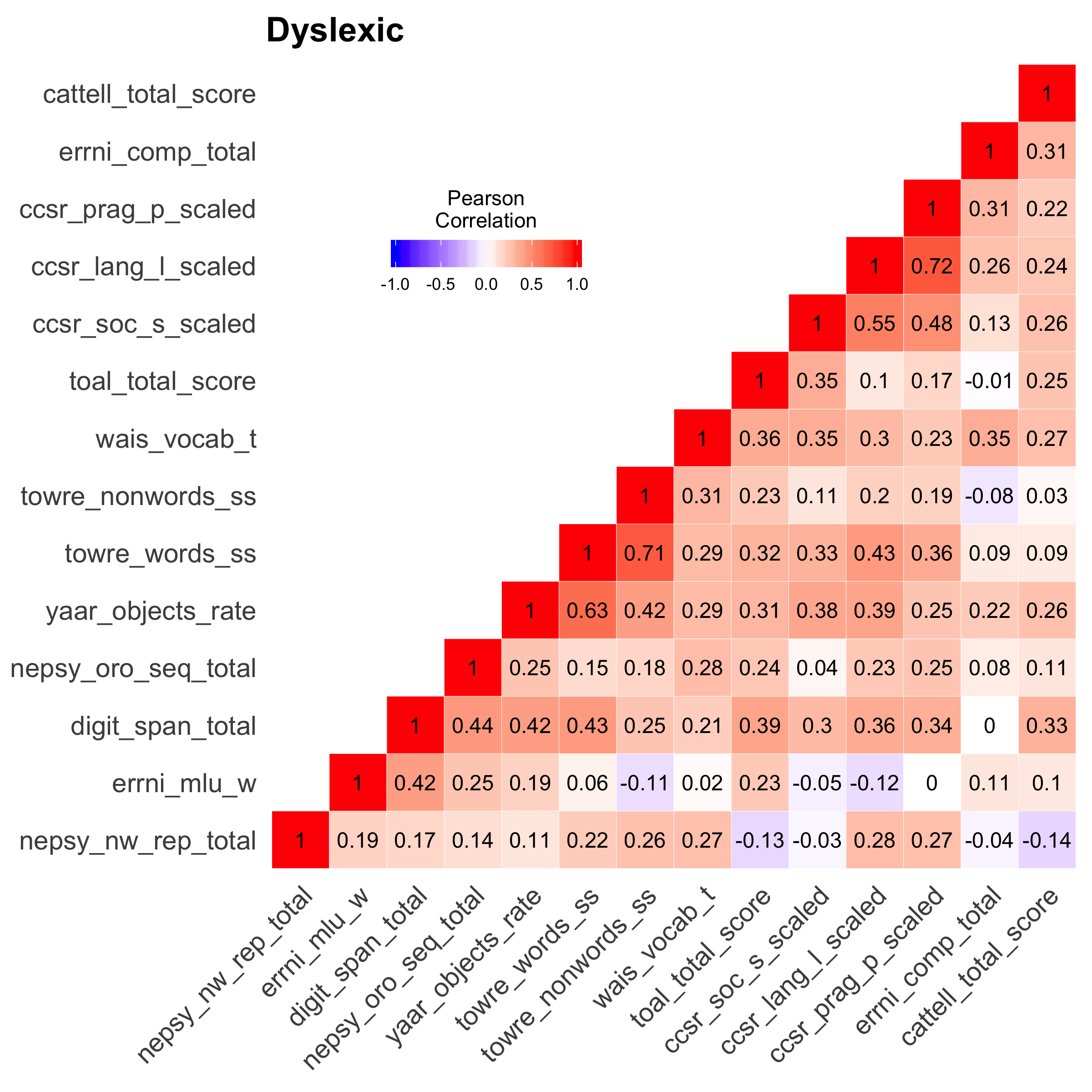

Supplement: Supplementary file 1 — Data S1 Supporting information [file DYS-27-3-s001.zip › DYS_1672_Appendix 2b.png]

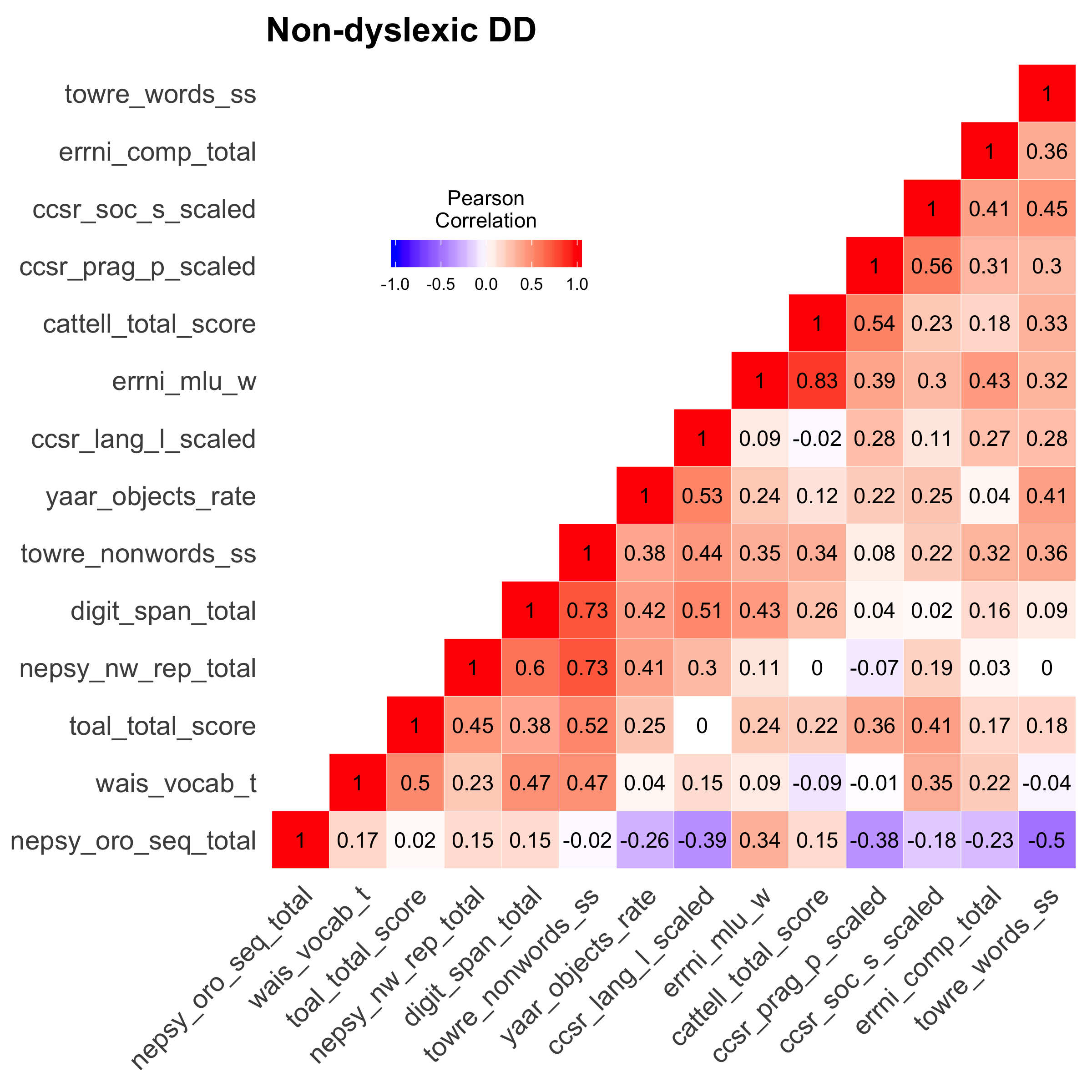

Supplement: Supplementary file 1 — Data S1 Supporting information [file DYS-27-3-s001.zip › DYS_1672_Appendix 2c.png]
